# Point-of-Care Ultrasound Diagnosis of Left Ventricular Thrombus: A Case Series

**DOI:** 10.7759/cureus.87192

**Published:** 2025-07-02

**Authors:** Tyler H Wen, Alina Mitina, Di Coneybeare

**Affiliations:** 1 Department of Emergency Medicine, Columbia University Vagelos College of Physicians and Surgeons, New York, USA

**Keywords:** cardiomyopathy, left ventricular aneurysm, left ventricular thrombus, point-of-care ultrasound, thromboembolic events

## Abstract

Left ventricular thrombus is a possible complication of heart failure, acute ischemic myocardial infarction, or progressive nonischemic cardiomyopathy. Early diagnosis of left ventricular thrombus can prevent end-organ ischemic events; however, it is complicated by the lack of easily accessible diagnostic modalities such as cardiac magnetic resonance imaging in the emergency department (ED). Here we present a case series of three patients who had point-of-care ultrasound (POCUS) evidence of left ventricular thrombus in the ED, which provided the emergency physician with valuable information that could be communicated to consulting and admitting teams. The case series illustrates the various common pathologic presentations associated with left ventricular thrombus: non-ischemic cardiomyopathy, ischemic cardiomyopathy, and sequelae of end-organ dysfunction such as cerebrovascular occlusions. Early recognition of left ventricular thrombus through POCUS can expedite subsequent management, and further studies are needed to demonstrate its effect on patient morbidity and mortality.

## Introduction

Left ventricular (LV) thrombus is a highly morbid condition that affects upwards of 25% of patients who experience anterior ST-segment elevation myocardial infarctions (STEMIs) and chronic apical LV infarcts, and 2-36% of patients with non-ischemic cardiomyopathies [1,2]. The variability seen in non-ischemic cardiomyopathies reflects the variability of clinical suspicion, the frequency and timing of screening modalities, and the efficacy of each screening modality [2]. 

The presence of an LV thrombus can significantly increase the risk of systemic embolization events like ischemic stroke, and is also associated with increased risk of major adverse cardiovascular events [1]. As such, early diagnosis and initiation of anticoagulation can help reduce the risk of adverse patient outcomes by halting the progression of further clot development. However, it can be difficult to obtain diagnostic modalities such as transthoracic echocardiography (TTE) or cardiac magnetic resonance imaging (cMRI) in the emergency department (ED) due to resource constraints. Point-of-care ultrasound (POCUS), a diagnostic ultrasound performed at the bedside by clinicians to answer specific clinical questions, is therefore an attractive method to potentially rule in left ventricular thrombus in an expedited fashion, especially as the evaluation of global systolic function is a routine aspect of cardiac POCUS.

Here, we present a case series of three patients who presented to the ED and were found to have evidence of LV thrombus in the ED through the use of POCUS and later confirmed through the use of other imaging modalities, which allowed the primary teams to rapidly obtain valuable diagnostic information and guide each patient’s ultimate management.

## Case presentation

Case 1

**Figure 1:**
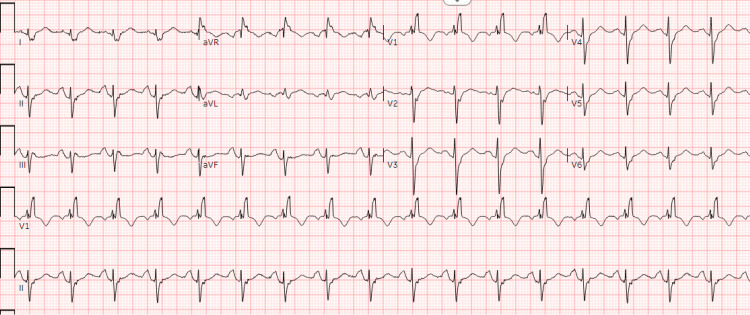
Electrocardiogram showing sinus tachycardia with a known right bundle branch block (Case 1)

An 83-year-old male patient with a past medical history significant for cirrhosis, hepatocellular carcinoma with metastases, prior history of cardiogenic shock, and non-ischemic cardiomyopathy complicated by LV thrombus, presented to the ED with three days of intermittent substernal chest pressure and nocturnal lightheadedness. His LV thrombus was first noted one month prior to this presentation, and he remained compliant on twice-daily dosing of apixaban. His vitals were notable for blood pressure of 112/77 mmHg, heart rate of 111 beats/minute, respiratory rate of 18 breaths/minute, temperature of 36.7°C, and an oxygen saturation of 97% on room air. His electrocardiogram (ECG) was notable for sinus tachycardia (Figure 1), and his laboratory workup was notable for a high-sensitivity troponin of 85 ng/L (reference <22 ng/L) that later down-trended to 79 ng/L (Table 1), raising concerns for an non-STEMI (NSTEMI).

**Table 1:** Troponin values in Case 1

	Patient Value	Reference Value
Initial Troponin	85 ng/L	< 22 ng/L
Second Troponin	79 ng/L	< 22 ng/L

**Figure 2:**
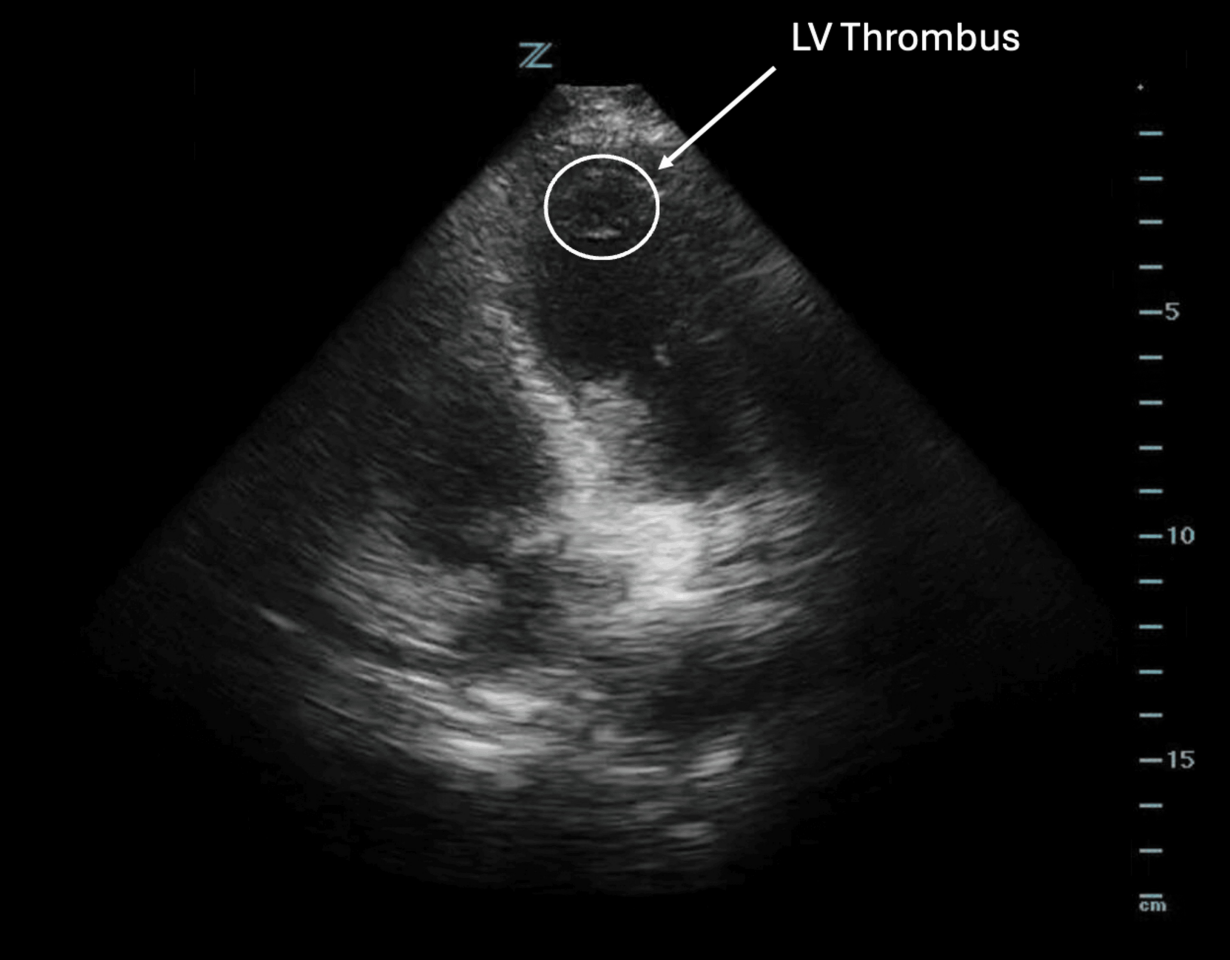
An apical four-chamber view of the left ventricle in Case 1, obtained using a phased array probe, demonstrating a left ventricular thrombus (outlined in white circle).

A bedside POCUS was performed and revealed persistence of his apical LV thrombus, which appeared as an echogenic intracardiac mass (Figure 2). This further raised concern for possible acute coronary sequelae of the LV thrombus, such as coronary artery emboli or NSTEMI, that would explain the patient's chest pain. No signs of right heart strain (such as right ventricular enlargement) were identified on POCUS, and the patient's lack of hypoxia was reassuring against pulmonary embolism as a differential. Given the POCUS findings and elevated troponins, the patient was admitted for further workup of his chest pain. A cardiology-performed TTE did not note any new concerning wall-motion abnormality and therefore did not lead to further intervention. He was ultimately discharged with continuation of apixaban indefinitely.

Case 2

A 70-year-old male patient with a past medical history significant for asthma, triple-vessel coronary artery disease, and a recent STEMI that was managed in the Dominican Republic two weeks prior, presented to the ED with chest pain. Per the patient, he had recently undergone a cardiac catheterization in the Dominican Republic, which led to the placement of two drug-eluting stents in his coronary arteries. There, he was admitted to the intensive care unit, where he was told he had severe heart failure. He had a right internal jugular central line placed and was offered open surgery for the management of his triple-vessel coronary artery disease. The patient decided, however, to return to the United States for further care.

**Figure 3:**
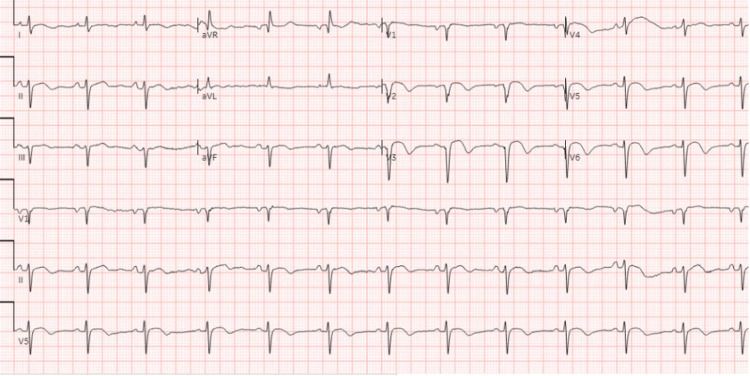
Initial electrocardiogram obtained in Case 2, notable for ST segment elevation in leads V2-V6 without reciprocal changes.

**Figure 4:**
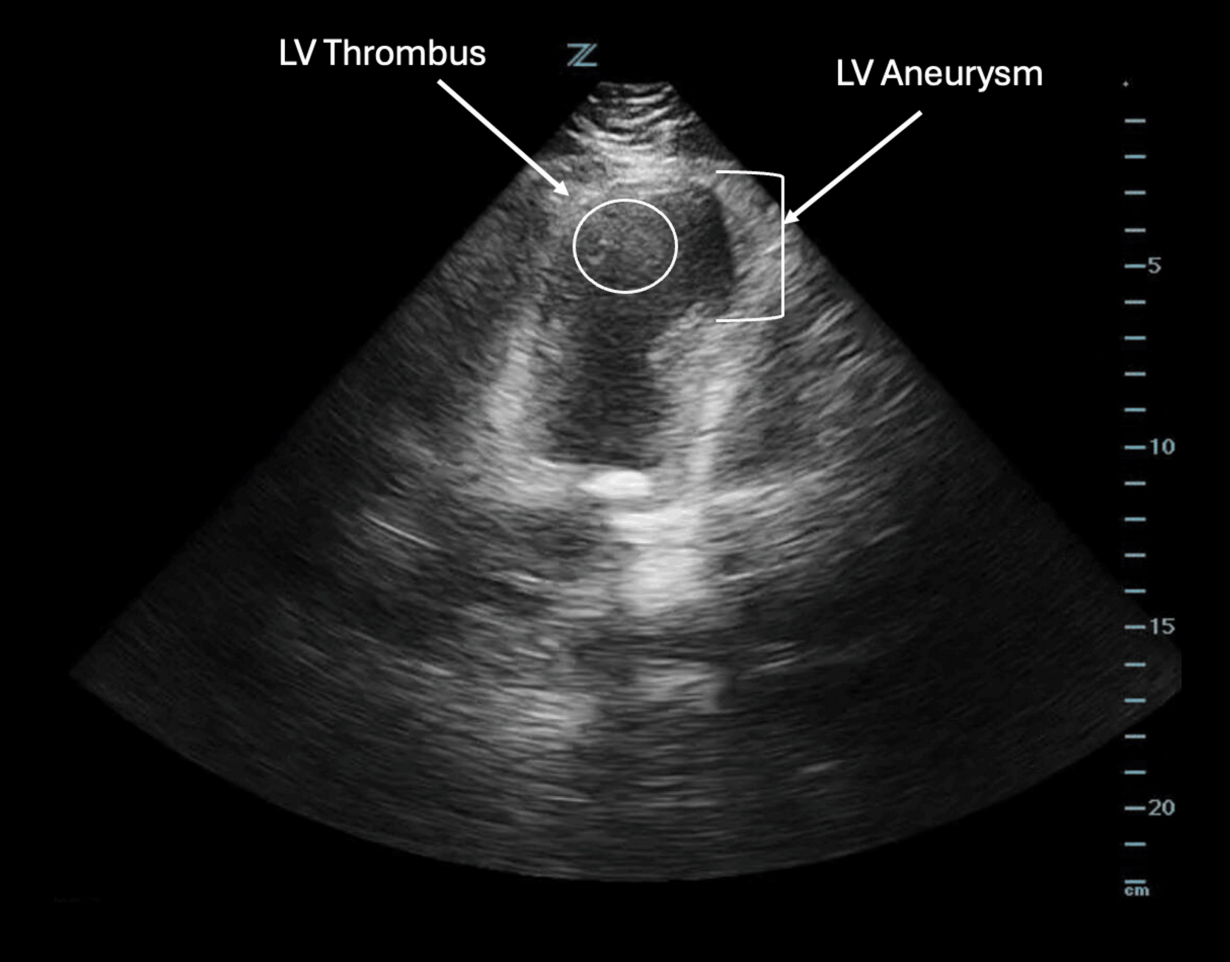
An apical four-chamber view of the left ventricle in Case 2, obtained using a phased array probe, demonstrating left ventricular aneurysm with a left ventricular thrombus (outlined in white circle).

His initial vitals were notable for blood pressure of 133/74 mmHg, heart rate of 83 beats/minute, respiratory rate of 18 breaths/minute, oxygen saturation of 98% on room air, and a temperature of 37.3 °C. His physical examination was notable for a right internal jugular triple-lumen catheter that was secured in place. An initial ECG was notable for ST-segment elevations in the precordial leads, with no prior ECG for comparison (Figure 3). Given concern for possible LV aneurysm, a bedside POCUS was performed and revealed the presence of an LV aneurysm and a subtle echogenic mass, raising suspicion for possible LV thrombus (Figure 4). Cardiothoracic surgery and cardiology were consulted, and based on these findings, the patient was started on a heparin bolus and infusion per their recommendations. He was then admitted to the cardiology service for further management. 

As an inpatient, the patient was able to provide his medical records from the Dominican Republic, which revealed that he suffered a 100% occlusion of his left anterior descending artery. He was not taking anticoagulation at the time of presentation. A cardiology-performed TTE two days after admission revealed a possible small early thrombus in the apex of the left ventricle, concordant with ED findings, and the patient was additionally started on aspirin and ticagrelor dual antiplatelet therapy. A diagnostic left heart catheterization was performed, which revealed patent stents in his left anterior descending artery. He was ultimately discharged 10 days after his initial ED visit on a regimen of apixaban, clopidogrel, and metoprolol. 

Case 3

**Figure 5:**
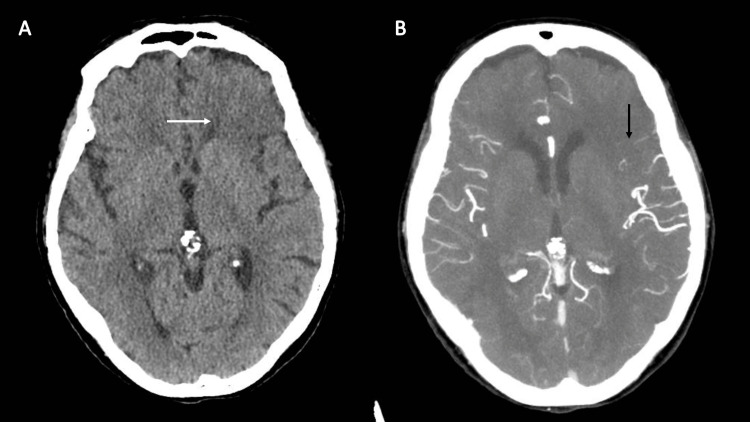
CT findings in Case 3 (A) Non-contrast head CT demonstrating subtle loss of gray-white differentiation in the left frontal cortex (white arrow); (B) CT angiography demonstrating abrupt cutoff in the superior left M2 distribution (black arrow).

An 87-year-old female patient with a past medical history significant for reflux esophagitis, hypertension, hyperlipidemia, chronic kidney disease, and rectal cancer presented to the ED with an acute change in mental status with a last known well time of roughly six hours prior to arrival. Vitals were notable for blood pressure of 119/67, heart rate of 70 beats/minute, respiratory rate of 19 breaths/minute, oxygen saturation of 100% on room air, and a temperature of 36.8 °C; fingerstick glucose was 100 mg/dL. On examination, she was only oriented to self, had minimal and hesitant speech, and had a degree of mixed receptive and expressive aphasia. A stroke code was called, and a non-contrast head computed tomography (CT) revealed findings concerning for an acute stroke in the left frontal cerebral cortex, while a CT angiography was suggestive of a middle cerebral artery occlusion (Figure 5).

**Figure 6:**
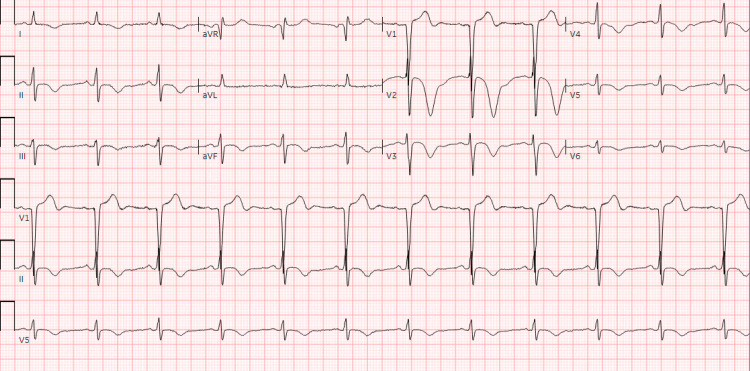
Electrocardiogram in Case 3 revealing inverted T waves in leads V2-6, along with very subtle STE in aVR

While neurology consultants were discussing risks and benefits of possible thrombectomy with their neurosurgery colleagues, the patient received an electrocardiogram, which revealed a subtle elevation in lead aVR and diffuse depressions in multiple other leads, leading the ED team to activate a code STEMI (Figure 6).

**Figure 7:**
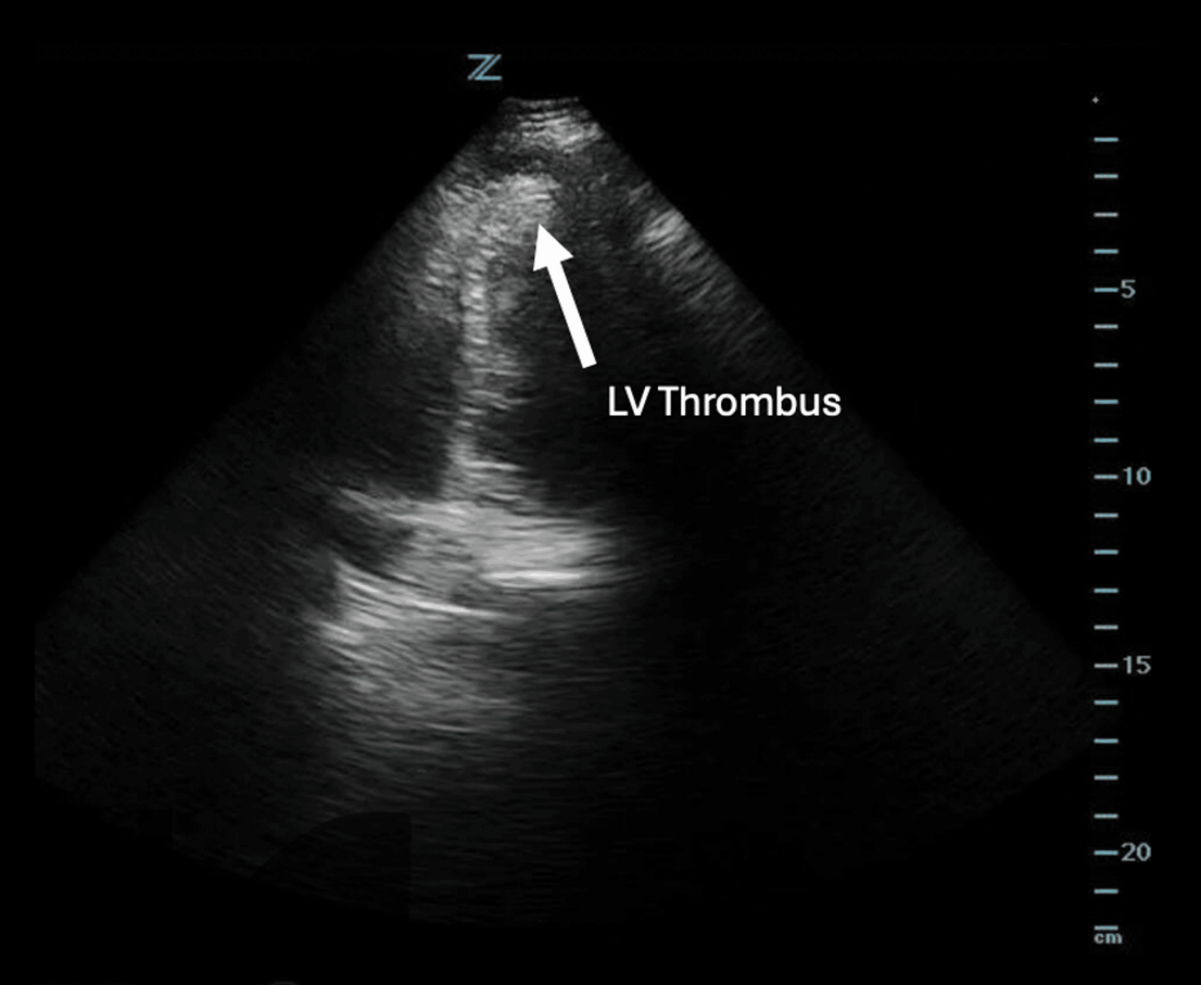
An apical four-chamber view of the left ventricle in Case 3, obtained using a phased array probe, demonstrating a small echogenic mass that is concerning for left ventricular thrombus.

A cardiac POCUS examination was performed at the bedside to look for regional wall motion abnormalities at this time. However, it discovered grossly decreased ejection fraction, apical ballooning, and possible evidence of an LV thrombus (Figure 7). Subsequently, high-sensitivity troponin test showed 315 ng/L (reference: < 22 ng/L), and pro-B-type natriuretic peptide (pro-BNP) resulted at 15,186 ng/L (baseline three months ago of 153 ng/L) (Table 2). Cardiology evaluated the patient and felt that her overall clinical picture and laboratory findings were representative of stress cardiomyopathy and not of an acute occlusive myocardial infarction, and she was ultimately admitted to the neurology stroke service for further workup. Pharmacologic and surgical treatment options were deferred at this time due to the patient's age and discussion of goals-of-care with family pending more comprehensive diagnostic testing. 

**Table 2:** Laboratory findings in Case 3 pro-BNP: pro-B-type natriuretic peptide

	Patient Value	Reference Value
Troponin	315 ng/L	< 22 ng/L
Glucose	100 mg/dL	75 - 100 mg/dL
pro-BNP	15,186 ng/L	< 624 ng/L

**Figure 8:**
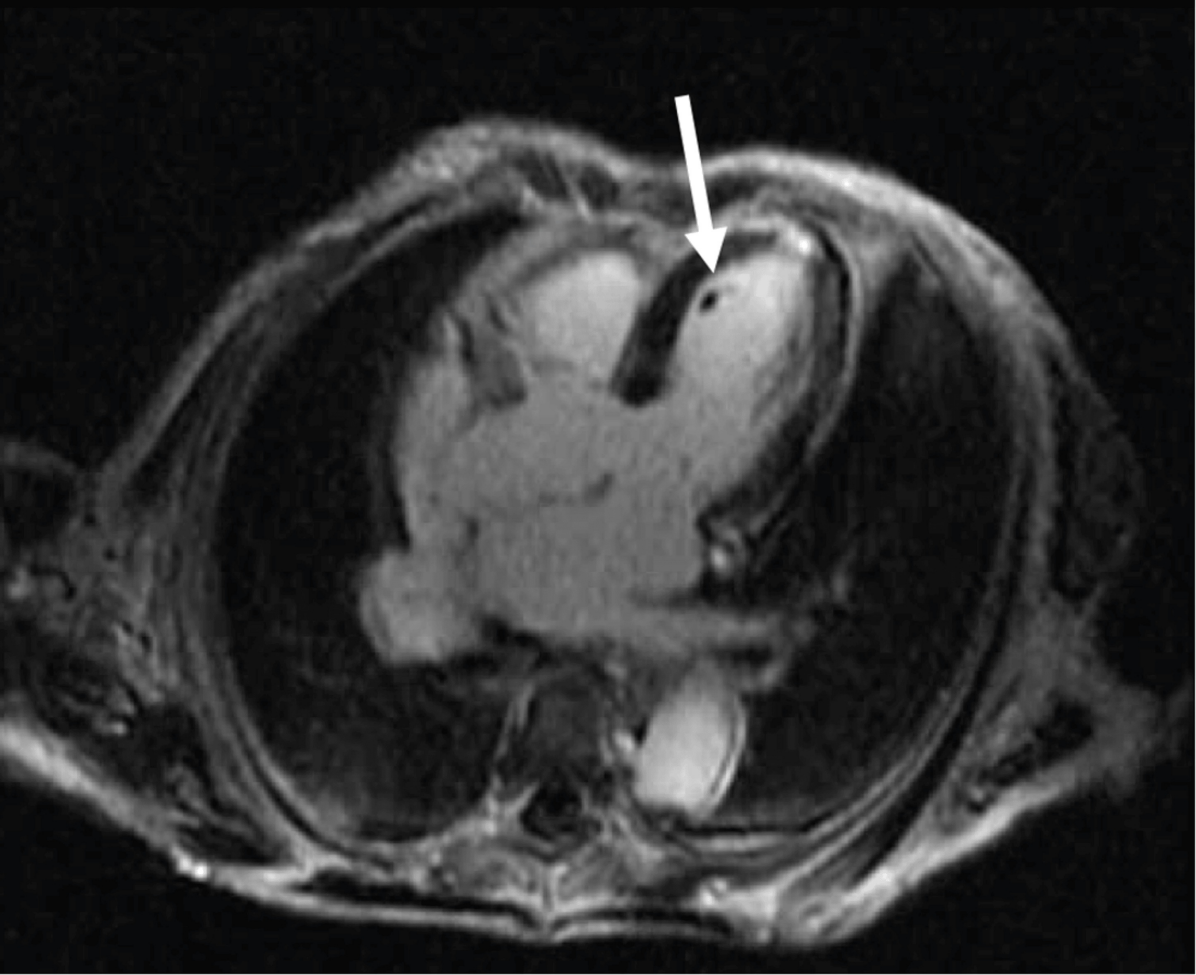
Cardiac magnetic resonance imaging in Case 3, revealing a discrete thrombus (white arrow) within the left ventricle.

As an inpatient, the patient received a cardiology-performed TTE with an estimated ejection fraction of 35-40%, and it could not exclude an LV thrombus. On hospital day 2, a cMRI confirmed initial POCUS suspicions and revealed a 0.6cm LV thrombus along with evidence of multiple myocardial infarcts that were likely embolic in origin (Figure 8). The LV thrombus was therefore likely the cause of the patient's acute stroke. The patient was started on a heparin infusion and ultimately transitioned to therapeutic-dosing apixaban upon discharge 12 days later. She was also started on oral metoprolol for her new diagnosis of heart failure with reduced ejection fraction, likely secondary to cardiomyopathy, given MRI findings. 

## Discussion

In this case series, we highlight the utility in looking for POCUS evidence of LV thrombus in patients who present to the ED with risk factors such as severe cardiomyopathy, LV aneurysm, or recent myocardial infarction (MI). The first case concerns a patient with nonischemic cardiomyopathy who had a recent LV thrombus; a bedside POCUS helped recognize the patient’s ongoing active thrombus burden, which raised suspicion for a thromboembolic etiology of the patient's chest pain, and helped strengthen the need for admission with the inpatient team. The second case concerns a patient with recent MI who was found to have evidence of an LV aneurysm on electrocardiogram, and a subsequent bedside POCUS helped identify both the aneurysm as well as an LV thrombus to expedite initiation of therapeutic anticoagulation. Studies have shown LV thrombus to develop mostly within the first two weeks post MI [3]. Finally, the third case concerns a patient with likely stress cardiomyopathy who presented to the ED with sequelae of LV thrombosis (i.e., ischemic stroke), where a bedside POCUS revealed evidence of an LV thrombus, which was ultimately the likely source of the cerebral emboli. 

LV thrombus is a highly morbid condition that ED physicians are well aware of, as an untreated LV thrombus can lead to devastating systemic thromboembolic events such as ischemic stroke, mesenteric ischemia, or critical limb ischemia [4,5]. As such, early identification and treatment are key to reducing patient morbidity and mortality, but diagnosis is often delayed due to difficulties in obtaining high-sensitivity cardiac imaging such as a cMRI, contrast-enhanced TTE, or transesophageal echocardiography (TEE). Screening for LV thrombus typically begins with TTE, which, depending on the inciting etiology of the LV thrombus, may have sensitivities ranging from 21-29% (for post-MI patients) to ~94% (for severe cardiomyopathy) due to variations in clot size and specific myocardial segments affected [3,6]. cMRI is a significantly more sensitive diagnostic modality in the diagnosis of post-MI LV thrombus, with sensitivities of 82-88% when compared to invasive surgical or pathological confirmation [3]. TEE and contrast cardiac CT are also more sensitive options for the diagnosis of LV thrombus; however, these modalities, along with cMRI, are all very difficult to obtain in the ED setting due to resource constraints and are not routinely obtained within the ED. This leaves TTE as the primary way in which emergency physicians can diagnose LV thrombus in the ED. While POCUS can be a powerful early diagnostic modality for ED physicians, its utility is limited by operator skill and comfort with POCUS applications. However, POCUS may serve as a valuable rule-in diagnostic modality, and emergency physicians should maintain a high index of suspicion for LV thrombus in patients with cardiomyopathy, warranting focused assessment for its presence.

**Figure 9:**
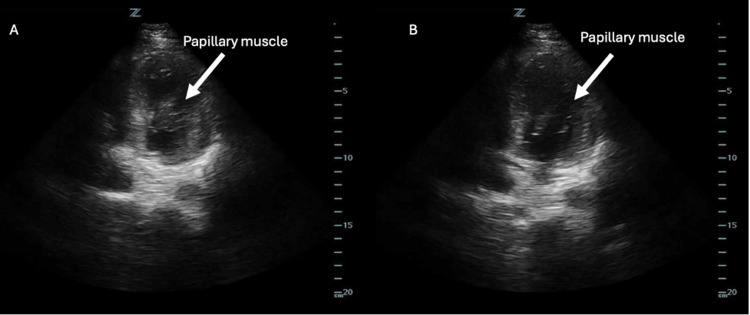
(A) An apical four-chamber view of the left ventricle obtained using a phased-array probe, which shows a papillary muscle that appears free-floating and may be misinterpreted as a thrombus; (B) The same apical four-chamber view, now revealing that the papillary muscle is connected to the ventricular wall. Image Credit: Original images by authors

On POCUS, an LV thrombus typically appears as a mobile, echogenic mass within the LV chamber. Note that an LV thrombus may be mistaken for prominent papillary muscles, as they both contain similar echogenicity. However, a papillary muscle can be distinguished by its attachment to the ventricular walls, while an LV thrombus is free-floating (Figure 9). It is therefore wise to visualize the heart in multiple views, such as the parasternal long, parasternal short, or axial four-chamber view, as the endocardial base of a papillary muscle may not be readily apparent in a single view.

Other differentials for echogenic masses within the LV include cardiac tumors and vegetation. Tumors will often have the presence of color flow on POCUS, whereas vegetation and thrombus will not [7]. The differentiation between vegetation and thrombus may be more subtle. However, thrombus formation occurs in the setting of low-flow states, and therefore LV thrombi typically are found in areas of significant regional wall motion hypokinesis, such as LV aneurysms. Vegetations, meanwhile, are typically found attached to valvular leaflets, such as in infective endocarditis, where areas exposed to high-flow states tend to attract more bacterial burden. Additionally, the overall clinical picture is important to contextualize POCUS findings that could raise suspicion for an LV thrombus, as an echogenic mass within the LV chamber presents different differentials for a patient with a recent STEMI and subsequent LV aneurysm, as opposed to a patient with months of weight loss and a new murmur. Ultimately, confirmatory diagnostics such as contrast-enhanced TTE or cardiac MRI can further differentiate the nature of LV masses on an inpatient basis.

## Conclusions

This case series highlights the utility of POCUS in facilitating bedside identification of LV thrombus in patients who present with concerning risk factors, such as severe heart failure or recent myocardial infarctions, or clinical features, such as acute stroke in the setting of severe cardiomyopathy. Bedside POCUS can be performed within minutes, poses no additional cost or radiation risk to the patient, and can provide clinically valuable information much faster than waiting for radiology-performed or cardiology-performed imaging studies. These cases provide examples of the benefits of early recognition of LV thrombus, as bedside POCUS can identify patients who are at high risk for complications, expedite specialist consultation, and ultimately lead to early initiation of anticoagulation, as an undiagnosed LV thrombus can lead to severe end-organ ischemic events. Upon identification of POCUS findings suspicious for an LV thrombus, it is therefore important to pursue additional confirmatory diagnostic modalities like TTE or cMRI, and confer with cardiology consultants. In this case series, cardiology-performed TTE studies and radiology-performed cMRI studies were concordant with bedside POCUS findings in identifying LV thrombus. While further studies would be needed to determine if early initiation of anticoagulation for LV thrombus carries clinically meaningful outcomes, ED physicians should consider performing bedside POCUS in patients they suspect of having an LV thrombus. 
